# Adult Hepatic Infarction for Internists: A Practical Diagnostic and Management Pathway to Avoid Misdiagnosis, Unnecessary Drainage, and Delayed Vascular Recognition

**DOI:** 10.3390/healthcare14142116

**Published:** 2026-07-15

**Authors:** Daniela Tirotta, Paolo Muratori

**Affiliations:** 1 Department of Internal Medicine, Morgagni-Pierantoni Hospital, AUSL Romagna, 47121 Forlì, Italy; paolo.muratori@auslromagna.it; 2 Department of Medical and Surgical Sciences, University of Bologna, S. Orsola-Malpighi Hospital, 40138 Bologna, Italy

**Keywords:** hepatic infarction, internal medicine, focal liver lesions, diagnostic reasoning, patient safety, sepsis, shock, contrast-enhanced ultrasound, computed tomography, magnetic resonance imaging, vascular liver disease, multidisciplinary care

## Abstract

**Introduction:** Hepatic infarction is an uncommon but clinically important cause of focal ischemic liver injury. In internal medicine, it usually emerges in acutely ill or complex hospitalized adults with sepsis, shock, malignancy, thrombosis, vasculitis, transplantation, or recent hepatobiliary or interventional procedures who develop a new focal hepatic lesion and abnormal liver tests. **Aims:** This review aims to provide internists with a practical diagnostic and management pathway for recognizing hepatic infarction, distinguishing it from abscess and malignancy, selecting appropriate imaging, identifying vascular complications, and avoiding unnecessary biopsy or drainage when conservative management is safer. **Methods:** We performed a structured narrative review of adult hepatic infarction and related ischemic liver entities using targeted searches in PubMed/MEDLINE and Scopus. The search strategy, screening process, and evidence limitations are reported explicitly. Because the available evidence is dominated by case reports, small series, radiology reviews, and guidance on related vascular liver diseases, the synthesis is qualitative and the proposed pathway is conceptual rather than prospectively validated. **Results:** Hepatic infarction is most often recognized when systemic hypoperfusion, splanchnic vasoconstriction, microvascular dysfunction, or local macrovascular compromise coexist. Multiphasic computed tomography (CT) is usually the first-line acute-care modality, while MRI, contrast-enhanced ultrasound, Doppler ultrasound, CT angiography, or vascular imaging should be selected according to diagnostic uncertainty and suspected arterial or portal venous complications. The main diagnostic pitfalls are misclassification as abscess, malignancy, hematoma, or postoperative collection. **Conclusions:** In the appropriate clinical context, a wedge-shaped or geographic non-enhancing hepatic lesion should trigger vascular-ischemic reasoning before being labeled as abscess or tumor. The proposed internist-led pathway is intended as a pragmatic conceptual framework for diagnostic reasoning and multidisciplinary communication, not as a validated guideline or evidence-based algorithm.

## 1. Introduction

Ischemic liver injury in adults encompasses a spectrum ranging from diffuse hypoxic liver injury to focal hepatic infarction. The term hypoxic liver injury is preferable to “hypoxic hepatitis” when the initiating event is circulatory or oxygen-delivery failure rather than primary inflammation. Diffuse hypoxic injury typically produces a rapid and often dramatic aminotransferase increase in the setting of cardiac failure, shock, respiratory failure, or severe systemic hypoxemia. By contrast, hepatic infarction refers to focal or geographic parenchymal necrosis caused by insufficient regional perfusion [1,2].

The liver is relatively resilient to transient reductions in blood flow because it receives inflow from both the portal vein and the hepatic artery and because the hepatic arterial buffer response can partially compensate for reductions in portal flow [3,4]. For this reason, focal infarction is uncommon and usually requires prolonged systemic hypoperfusion, local macrovascular compromise, microvascular thrombosis, or a combination of these mechanisms. Although historical clinicopathologic studies remain foundational, contemporary discussion of hepatic infarction should be framed through modern concepts of acute-care imaging, sepsis-associated microvascular dysfunction, portal venous thrombosis, iatrogenic vascular injury, and patient safety [5,6,7,8,9] (Table 1).

The clinical relevance of hepatic infarction is disproportionate to its frequency. A peripheral non-enhancing hepatic lesion in a patient with sepsis, shock, cancer, recent hepatobiliary intervention, transplantation, thrombophilia, or vascular disease may be mistaken for abscess or malignancy [10,11,12,13]. This error may expose fragile patients to biopsy, drainage, surgery, prolonged antimicrobial therapy, repeated cross-sectional imaging, and avoidable care escalation. For a healthcare journal, hepatic infarction is therefore not only a rare clinicopathologic entity; it is also a diagnostic pathway and patient-safety problem at the interface of acute medicine, imaging, multidisciplinary decision-making, and resource stewardship.

The aim of this review is to give internists a practical diagnostic and management framework for a difficult real-world scenario: a complex hospitalized adult with a new focal hepatic lesion. The review focuses on when to suspect hepatic infarction, how to distinguish it from abscess and malignancy, when to request computed tomography (CT), magnetic resonance imaging (MRI), contrast-enhanced ultrasound (CEUS), or vascular imaging, when to involve other specialists, and when conservative management is preferable to biopsy or drainage.

## 2. Methods

Search strategy. This article is a structured narrative review. A targeted literature search was performed in PubMed/MEDLINE and Scopus for adult hepatic infarction and related ischemic liver entities published between January 2000 and December 2025. Selected pre-2000 landmark clinicopathologic, imaging, or pathophysiology sources were retained through backward citation checking only when they provided foundational information that remains clinically relevant and could not be replaced by newer evidence. The following core search strings were used: (“hepatic infarction” OR “liver infarction” OR “focal hepatic ischemia”); (“hepatic infarction” AND (“sepsis” OR “shock” OR “hypoxic liver injury”)); (“hepatic infarction” AND (“hepatic artery thrombosis” OR “hepatic artery pseudoaneurysm” OR “portal vein thrombosis”)); (“hepatic infarction” AND (“computed tomography” OR “magnetic resonance imaging” OR “contrast-enhanced ultrasound”)); (“hepatic infarction” AND (“acute pancreatitis” OR “pylephlebitis” OR “septic portal vein thrombosis”)); and (“ischemic cholangiopathy” AND (“hepatic artery” OR “transplantation”)). Additional targeted searches were performed for sepsis-associated endothelial dysfunction, immunothrombosis, hepatic arterial buffer response, and Toll-like receptors (TLRs) in liver regeneration. The reporting structure was strengthened using PRISMA-style flow reporting and narrative-review quality principles, including transparent search description, explicit study selection, distinction between evidence-supported statements and expert-opinion synthesis, and balanced discussion of limitations.

Eligibility and selection. We included English-language adult case reports, case series, retrospective cohorts, radiology reviews, hepatology guidance documents, interventional radiology reports, and the transplant-related literature when they informed pathophysiology, diagnosis, differential diagnosis, management, follow-up, or multidisciplinary care. Pediatric reports, animal-only studies not relevant to the requested mechanistic discussion, non-hepatic infarction reports, duplicate publications, conference abstracts without sufficient clinical detail, and articles not addressing focal hepatic ischemia or directly relevant mimics were excluded. Earlier landmark clinicopathologic and radiologic studies were retained only when they provided foundational information that remains clinically relevant.

**Figure 1:**
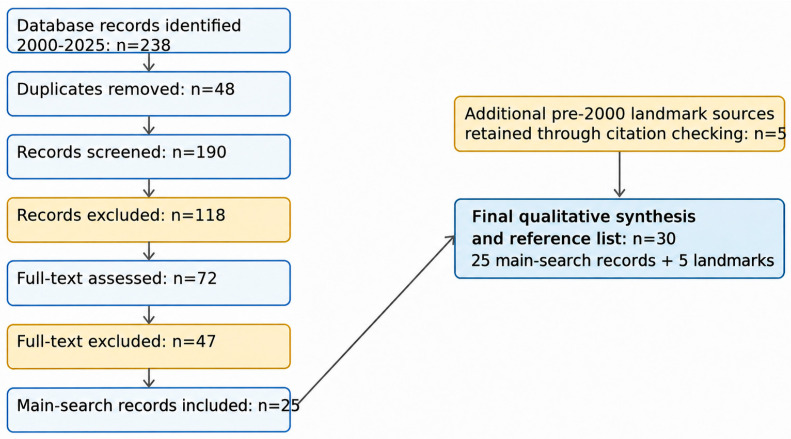
PRISMA-style flow of the targeted narrative review: database records identified for January 2000–December 2025 (n = 238); duplicates removed (n = 48); records screened (n = 190); records excluded after title/abstract screening (n = 118); full-text articles assessed from the main search (n = 72); full-text articles excluded (n = 47); contemporary records included from the main search (n = 25); additional pre-2000 landmark sources retained through citation checking (n = 5); final studies/guidance documents included in qualitative synthesis and cited in the reference list (n = 30). Counts should be interpreted as the transparent flow of a targeted narrative review rather than as a systematic review meta-analysis.

Screening flow. The database search identified 238 records published between January 2000 and December 2025 (PubMed/MEDLINE, n = 142; Scopus, n = 96). After removal of 48 duplicates, 190 records were screened by title and abstract. One hundred and eighteen records were excluded because they were pediatric, non-hepatic, unrelated to focal hepatic ischemia, duplicate case descriptions, conference-only material, or not clinically relevant to the review question. Seventy-two full-text articles from the main search were assessed for eligibility; 47 were excluded because they provided insufficient clinical detail, focused on unrelated ischemic syndromes, or did not add actionable diagnostic or management information. Twenty-five contemporary records from the main search were included. Five additional pre-2000 landmark sources were retained through citation checking for historical clinicopathologic, imaging, or hepatic arterial buffer response context. Therefore, 30 studies and guidance documents were included in the qualitative synthesis and cited in the reference list (Figure 1).

Evidence synthesis and wording of recommendations. Because the evidence base is dominated by case reports, small series, imaging reviews, and extrapolation from related vascular liver disease guidance, no pooled incidence, pooled diagnostic accuracy estimate, formal risk-of-bias assessment, or meta-analysis was attempted. Throughout the manuscript, statements directly supported by the published literature are distinguished from expert-opinion synthesis and are phrased cautiously. Expert-opinion synthesis was used only to translate fragmented case-based evidence into practical bedside reasoning and is explicitly identified as such. The proposed pathway is conceptual and intended as a bedside framework for internists; it has not undergone prospective validation and should not be interpreted as a guideline.

## 3. Epidemiology and Under-Recognition

Reliable epidemiologic data are scarce. Hepatic infarction is primarily described in autopsy studies, radiology series, transplant populations, vascular complication reports, and isolated case reports. In a CT-based radiology series of patients investigated for acute abdominal pain, hepatic infarction was reported in approximately 0.2–0.4% of scanned cases; this denominator refers to selected patients undergoing CT for acute abdominal pain and cannot be generalized to unselected medical inpatients, intensive-care populations, or patients who do not undergo cross-sectional imaging [10].

Historical autopsy series confirm that hepatic infarction is infrequent, but they also show that it usually occurs in patients with major systemic illness, cardiac disease, thromboembolic disease, severe hypotension, or disseminated intravascular coagulation [5,6]. The true clinical burden may be underestimated because acutely ill patients may die before diagnostic imaging is completed; small infarcts may be clinically silent; focal necrosis may be attributed to abscess, metastasis, trauma, or post-procedural change; and follow-up imaging is not always performed.

It is therefore more accurate to state that modern CT, MRI, CEUS, and vascular imaging increase the opportunity to recognize hepatic infarction in selected high-risk settings, rather than that the condition is universally becoming more frequent. Recognition is particularly important after liver transplantation, hepatobiliary or pancreatic surgery, embolization procedures, biliary interventions, septic shock, vasopressor-treated shock, and acute portal or hepatic arterial vascular events.

## 4. Pathophysiology: Macrovascular and Microvascular Mechanisms

Hepatic infarction develops when the protective redundancy of hepatic perfusion is overwhelmed. The portal vein provides most hepatic blood flow, whereas the hepatic artery contributes a smaller but oxygen-rich component [3,4]. When portal flow decreases, the hepatic arterial buffer response may increase arterial inflow; however, this compensation can fail during severe vasodilation, vasopressor-treated shock, arterial injury, thrombosis, advanced chronic liver disease, or microvascular obstruction.

Macrovascular infarction results from interruption or severe reduction in hepatic arterial or portal venous flow. Causes include hepatic artery thrombosis, embolism, dissection, pseudoaneurysm, portal vein thrombosis, traumatic vascular injury, transplant-related arterial thrombosis, and post-embolization ischemia. A purely arterial lesion is more likely to produce clinically relevant necrosis when portal inflow is impaired or when the biliary tree is involved, because the bile ducts are particularly dependent on arterial blood supply.

Microvascular infarction is increasingly relevant in internal medicine and intensive-care practice. Sepsis and septic shock can induce endothelial dysfunction, leukocyte and platelet activation, immunothrombosis, disseminated intravascular coagulation, sinusoidal stasis, and regional hypoperfusion [7,8,9]. In this setting, macroscopic hepatic artery and portal vein patency does not exclude ischemic necrosis. Severe splanchnic vasoconstriction during shock and vasopressor therapy may further reduce effective perfusion.

**Figure 2:**
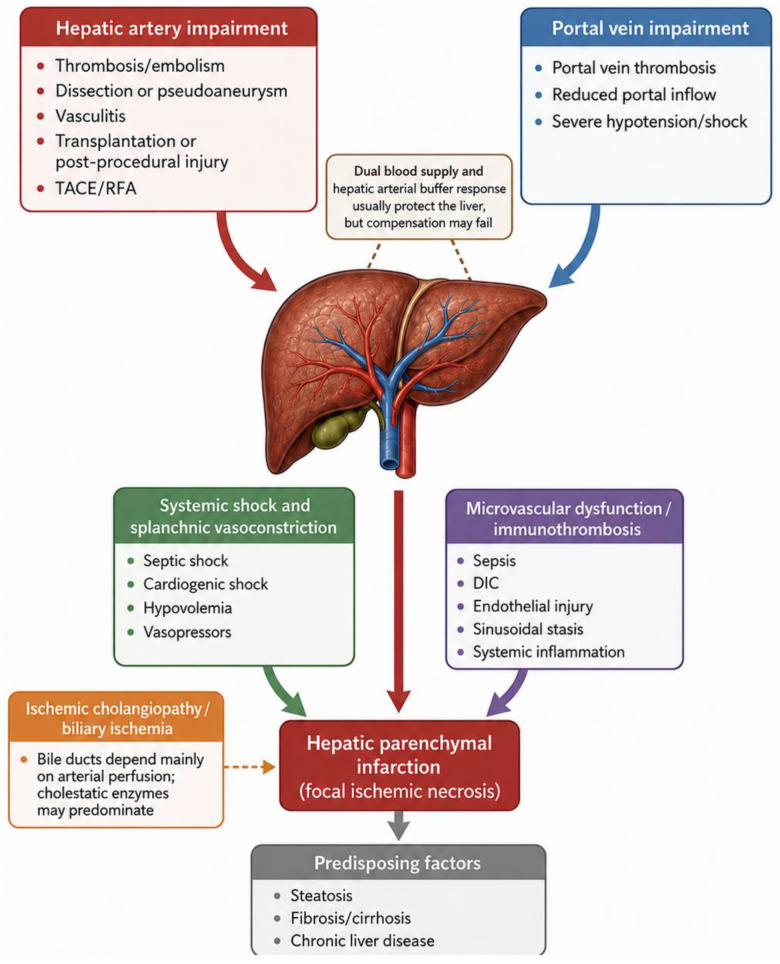
Pathophysiology of hepatic infarction in adults. The figure summarizes arterial, portal venous, systemic shock, biliary ischemic, and microvascular mechanisms contributing to focal ischemic hepatic necrosis.

Ischemic cholangiopathy deserves specific attention. In liver transplantation, hepatic artery thrombosis is a recognized vascular complication, and biliary ischemia may be an early or dominant manifestation because the bile ducts depend on arterial perfusion. Consequently, cholestatic enzyme elevation, especially gamma-glutamyl transferase and alkaline phosphatase, may precede or outweigh aminotransferase elevation in some arterial complications. The same principle applies, although less systematically, to severe shock or iatrogenic arterial injury outside transplantation (Figure 2).

## 5. Etiologic Settings

Hepatic infarction is best understood as a final common pathway rather than a single disease. Major etiologic settings include systemic shock and sepsis, hepatic artery thrombosis or occlusion, iatrogenic vascular injury, portal vein thrombosis, hypercoagulable states, systemic vasculitis, trauma, transplantation, and less common postoperative or endovascular/interventional arterial injuries [14,15].

The etiologic categories used in Table 2 are aligned with the main text and are intentionally organized by clinical scenario rather than by a single anatomic vessel. This approach is more useful for healthcare professionals because many patients have overlapping mechanisms. For example, septic shock may produce microvascular thrombosis and systemic hypoperfusion, while a recent biliary or hepatic intervention may add local arterial injury or pseudoaneurysm formation [7,8,9,16,17,18,19].

Iatrogenic causes deserve emphasis from a healthcare-systems perspective. Hepatobiliary surgery, pancreatic surgery, liver transplantation, percutaneous biopsy, transarterial embolization or chemoembolization, radiofrequency ablation, and endoscopic or radiologic biliary procedures can all injure arterial or portal venous structures. In these settings, the key clinical question is not merely whether an infarct is present, but whether an actionable complication such as arterial thrombosis, pseudoaneurysm, infected necrosis, bile duct ischemia, or portal vein thrombosis requires urgent intervention (Table 2).

## 6. A Practical Diagnostic Pathway for Internists

No major hepatology, radiology, internal medicine, or healthcare-management guideline provides a dedicated diagnostic pathway for adult hepatic infarction. Therefore, the internist must integrate three domains in real time: the bedside context, the vascular anatomy, and the imaging phenotype of the lesion. This review proposes a practical sequence rather than a rigid rule, because the evidence base is rare-disease- and case-based.

The first step is bedside probability. The diagnosis becomes plausible when a new focal hepatic lesion occurs in a patient with shock, sepsis, vasopressor exposure, acute thromboembolism, atrial fibrillation, cancer-associated thrombosis, vasculitis, antiphospholipid syndrome, liver transplantation, or recent hepatobiliary/interventional procedures. Abrupt aminotransferase elevation, high lactate dehydrogenase (LDH), lactate elevation, cholestatic abnormalities, or unexplained coagulopathy increases the need for rapid clinical-imaging correlation.

The second step is lesion characterization. A multiphasic contrast-enhanced computed tomography (CT) scan is usually the most accessible first-line test in acute care. Magnetic resonance imaging (MRI) is useful when malignancy, abscess, or biliary ischemia remains uncertain. Contrast-enhanced ultrasound (CEUS) may be useful at the bedside, for renal impairment, for radiation-free follow-up, or when rapid vascular-phase assessment is needed. Vascular imaging should be considered when arterial thrombosis, pseudoaneurysm, dissection, portal vein thrombosis, or post-transplant vascular complications are possible.

The third step is management triage. Typical infarction in a stable patient can often be managed conservatively with treatment of the underlying cause and planned follow-up. Invasive procedures should be reserved for cases with diagnostic uncertainty after adequate imaging, suspected malignancy, persistent sepsis, secondary infection, enlarging collection, active bleeding, pseudoaneurysm, biliary ischemia, or treatable vascular occlusion. The pathway is designed to improve communication between internists, radiologists, intensivists, hepatologists, interventional radiologists, surgeons, microbiologists, and transplant teams.

## 7. Imaging Features, Pitfalls, and Do-Not-Miss Differentials

Imaging is central because symptoms and laboratory findings are nonspecific (Table 3). Patients may have right upper quadrant pain, fever, shock, leukocytosis, lactic acidosis, or marked aminotransferase and LDH elevation. Bilirubin may be normal initially, whereas cholestatic enzymes may become prominent when biliary ischemia develops. Imaging should answer four questions: Is the lesion non-enhancing? Is its morphology compatible with infarction? Is there a vascular cause? Is there evidence of infection, malignancy, hemorrhage, or another mimic?

Contrast-enhanced CT typically shows a peripheral, wedge-shaped, geographic, or segmental hypoattenuating area with absent or markedly reduced enhancement [10,11,12,13]. The lesion may extend to the capsule and may not respect a round mass-like contour. Early lesions can be subtle; later lesions may shrink, become more sharply demarcated, develop capsular retraction, or undergo liquefaction. Gas, rim-enhancing fluid collection, increasing fever, or persistent bacteremia should raise concern for secondary infection of infarcted tissue [16].

MRI can help when CT is inconclusive or when malignancy remains a concern. Reported features include T1 hypointensity, variable T2 hyperintensity, absent or reduced post-contrast enhancement, and restricted diffusion in necrotic or ischemic tissue [11,20,28]. Diffusion-weighted imaging should not be interpreted in isolation, because abscesses and tumors may also restrict diffusion. Features that favor infarction include wedge-shaped or geographic morphology, vascular-territory distribution, absence of an enhancing viable tumor rim or nodular enhancing tissue, compatible clinical context, and expected shrinkage or demarcation on follow-up. Features favoring abscess include gas, liquefaction, a drainable rim-enhancing collection, persistent bacteremia, or clinical sepsis despite source control; features favoring malignancy include mass effect, enhancing solid components, multifocal progressive growth, or tumor-specific clinical context.

CEUS provides real-time bedside vascular-phase assessment and is particularly useful when CT contrast is undesirable or when follow-up is needed without radiation [21,29,30]. A typical infarct appears as a sharply demarcated area of absent or persistent hypoenhancement during arterial, portal venous, and late phases. CEUS can also help distinguish avascular necrosis from a hypervascular tumor or a liquefied abscess, although deep lesions, obesity, bowel gas, and operator dependence are limitations.

The availability of CEUS is heterogeneous across healthcare systems, and its diagnostic performance depends on local expertise, acoustic window, lesion depth, body habitus, bowel gas, and the ability to integrate the examination with multiphasic CT or MRI. Where CEUS is unavailable or operator experience is limited, the practical alternative is careful review of multiphasic contrast-enhanced CT, Doppler ultrasound for vascular patency, MRI/MRCP when biliary ischemia or malignancy remains uncertain, and early radiology or hepatology discussion rather than reliance on a single imaging modality.

Common diagnostic pitfalls include interpreting fever and a non-enhancing lesion as abscess without considering ischemia; interpreting a peripheral wedge-shaped defect as metastasis; assuming that patent main vessels exclude microvascular infarction; overlooking hepatic artery thrombosis or pseudoaneurysm after procedures; and failing to recognize ischemic cholangiopathy when cholestasis predominates. For the internist, the practical discriminator is the combination of clinical setting, lesion geometry, enhancement behavior, vascular findings, and evolution over time. Imaging should be reviewed with radiology in light of the clinical trajectory, not interpreted in isolation (Table 3 and Table 4).

## 8. Acute Pancreatitis, Pylephlebitis, and Bland Portal Vein Thrombosis

A connection between acute pancreatitis and hepatic infarction is rare but biologically plausible and has been reported in the case literature. The proposed mechanisms include splenic or portal venous thrombosis, severe local inflammation, systemic inflammatory response, hypovolemia, vasoconstriction, endothelial injury, and pancreatitis-associated hypercoagulability [22]. In practice, hepatic infarction should be considered when a patient with acute pancreatitis develops a new peripheral hepatic lesion, abrupt liver enzyme deterioration, portal or splenic vein thrombosis, or persistent sepsis not explained by pancreatic necrosis alone.

Pylephlebitis, or septic thrombophlebitis of the portal venous system, is another uncommon but important context. It usually follows intra-abdominal infection such as diverticulitis, appendicitis, cholangitis, inflammatory bowel disease, or pancreatitis. Septic thrombus may reduce portal inflow, seed the liver, and produce hepatic abscesses; rarely, the dominant imaging manifestation can include hepatic infarction or infarction-like lesions, especially when portal obstruction is extensive or accompanied by arterial hypoperfusion [23,24]. In this setting, the management frame is not conservative infarction alone: source control, antibiotics, assessment for abscess, and individualized anticoagulation should be discussed.

Acute bland portal vein thrombosis (PVT) can also contribute to hepatic infarction when portal flow is abruptly reduced and compensatory arterial inflow is insufficient. This is more likely when PVT is extensive, involves intrahepatic branches, occurs with shock or arterial compromise, or develops in patients with malignancy, thrombophilia, pancreatitis, cirrhosis, or recent surgery. Current vascular liver disease guidance and expert updates emphasize prompt confirmation of the extent and acuity of PVT, assessment for intestinal ischemia and underlying prothrombotic disease, and individualized anticoagulation when benefits outweigh bleeding risk [17,18]. For the internist, the practical message is that portal venous thrombosis should not be considered merely an incidental imaging finding when a compatible non-enhancing hepatic lesion is present.

## 9. Prognosis, Outcomes, and Complications

Outcome depends less on the infarct itself than on its cause, extension, complications, and the severity of the underlying illness. Small peripheral infarcts may resolve or leave residual capsular retraction. Larger infarcts in the context of septic shock, cardiogenic shock, disseminated intravascular coagulation, transplant vascular complications, or multiorgan failure carry a worse prognosis. Patients who die during resuscitation or early intensive-care management may never receive a definitive diagnosis, which contributes to under-reporting.

Important adverse outcomes include secondary infection of infarcted parenchyma, abscess formation, persistent sepsis, biliary ischemia or ischemic cholangiopathy, liver failure in patients with limited reserve, and need for endovascular or surgical management when a vascular lesion is present [16,17,19]. Prognostic assessment should therefore include hemodynamic status, lactate, renal function, coagulation profile, extent of necrosis, biliary involvement, vascular patency, and clinical evolution over the first days.

## 10. Role of Liver Biopsy

Liver biopsy is rarely required when the clinical context and imaging pattern are typical. Histology classically shows coagulative necrosis with loss of hepatocyte architecture [5,6]. However, biopsy of infarcted or infected tissue may be nondiagnostic and can increase bleeding or infectious risk in acutely ill patients.

Biopsy should be reserved for selected cases in which malignancy, lymphoma, inflammatory pseudotumor, granulomatous disease, or atypical infection remains plausible after adequate contrast imaging and follow-up. Before biopsy, clinicians should review anticoagulation, platelet count, coagulation status, vascular anatomy, and whether a safer strategy such as short-interval imaging follow-up or drainage of a clearly infected collection would be more appropriate. Because biopsy of infarcted or infected tissue may be non-diagnostic, this statement should be interpreted as expert-opinion guidance based on the expected histologic heterogeneity and procedural risk in necrotic or infected hepatic tissue rather than on dedicated prospective diagnostic-accuracy data.

## 11. Clinical Management and Follow-Up

There is no disease-specific therapy for hepatic infarction; treatment targets the mechanism. The internist should stabilize perfusion and oxygenation, treat sepsis, correct severe hypovolemia, review vasopressor exposure, search for thrombotic or embolic sources, reassess anticoagulation when thrombosis is present, and identify iatrogenic vascular complications. Anticoagulation is not automatic: it should be considered when acute portal vein thrombosis, hepatic arterial thrombosis, embolic disease, antiphospholipid syndrome, or another treatable thrombotic mechanism is present, but it may be contraindicated or delayed in active bleeding, severe thrombocytopenia, uncontrolled coagulopathy, high-risk procedures, or hemorrhagic transformation. Decisions should be individualized and aligned with vascular liver disease guidance and multidisciplinary input. Antibiotics are indicated for sepsis, cholangitis, bacteremia, pylephlebitis, or secondary infection, but an uncomplicated infarct should not automatically be managed as an abscess.

Iatrogenic or structural vascular complications require early multidisciplinary discussion. Hepatic artery pseudoaneurysm, arterial dissection, active bleeding, post-transplant hepatic artery thrombosis, or procedure-related vascular occlusion may need interventional radiology, transplant surgery, vascular surgery, or hepatobiliary surgery [19].

Follow-up imaging should be tailored to severity and uncertainty. In stable patients with typical imaging and improving clinical status, repeat CT, MRI, or CEUS at approximately 4–6 weeks can document reduction in size, stable non-enhancement, or expected evolution. Earlier imaging is appropriate when symptoms worsen, liver tests deteriorate, fever or bacteremia persists, the lesion enlarges, gas appears, or a new rim-enhancing collection develops. CEUS is attractive for bedside follow-up when available, while MRI may be useful for persistent diagnostic ambiguity [21].

A practical management pathway should therefore include: (1) recognize the high-probability clinical scenario; (2) obtain or review multiphasic imaging; (3) assess arterial and portal venous patency and procedure-related vascular complications; (4) distinguish sterile infarction from secondary infection; (5) decide whether conservative management is safer than biopsy or drainage; (6) involve the appropriate specialist early when intervention may change outcome; and (7) schedule follow-up imaging to document expected evolution (Table 5 and Table 6).

## 12. Clinical Case and Imaging Correlation

The clinical case is an original unpublished illustrative observation from clinical practice, presented in anonymized form to show how the proposed reasoning pathway can be applied. It is not intended as a separate case report or as evidence of diagnostic accuracy. A middle-aged woman was admitted with septic shock due to Escherichia coli, with hypotension requiring acute resuscitation and broad-spectrum antimicrobial therapy. Laboratory assessment showed an acute hepatocellular injury pattern with marked aminotransferase and LDH elevation and initially limited cholestatic expression. Autoimmune and thrombophilic screening was negative. Clinical history included dyslipidemia and sporadic cannabinoid exposure.

**Figure 3:**
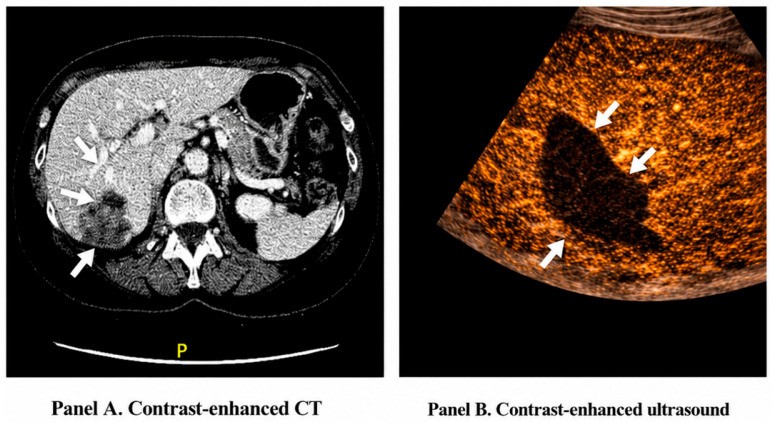
Hepatic infarction: CT and CEUS correlation in a real-world case involving segment VII. Panel (**A**): contrast-enhanced CT shows a peripheral wedge-shaped hypodense lesion in segment VII (arrows). Panel (**B**): contrast-enhanced ultrasound demonstrates an avascular, persistently hypoenhancing area corresponding to the infarcted region (arrows). Imaging interpretation is consistent with reported CT and CEUS features of hepatic infarction [10,21,29,30]. The CT and CEUS images are original anonymized clinical images from the Department of Internal Medicine, Morgagni-Pierantoni Hospital, AUSL Romagna, Forlì, Italy.

Contrast-enhanced CT demonstrated a peripheral wedge-shaped hypodense lesion in segment VII, without imaging features typical of a mass-forming tumor. CEUS showed absence of arterial enhancement and persistent hypoenhancement in the portal venous and late phases, supporting focal hepatic infarction rather than abscess or malignancy. CT angiographic assessment documented a small hepatic artery pseudoaneurysm rather than a generic microaneurysm; it was interpreted as a possible local contributor in the context of septic shock and microvascular dysfunction, but not as the sole proven cause of infarction. The pseudoaneurysm was subsequently embolized because of the recognized bleeding risk of hepatic arterial pseudoaneurysms and the need to treat an actionable vascular complication (Figure 3). The original CT and CEUS images used in Figure 3 were obtained from the Department of Internal Medicine, Morgagni-Pierantoni Hospital, AUSL Romagna, Forlì, Italy, after anonymization.

Management was initially conservative and directed toward sepsis control, hemodynamic stabilization, and imaging surveillance. No immediate biopsy or drainage was performed because the lesion morphology, vascular-phase behavior, and clinical context were consistent with infarction and there was no evidence of a drainable infected collection. After pseudoaneurysm embolization, serial CEUS follow-up documented the expected evolution of the lesion without features of progressive infection or malignancy. MRI was not pursued because CT and CEUS were concordant, the lesion had a typical vascular-ischemic pattern, the patient improved clinically, and follow-up CEUS showed expected evolution. MRI would have been appropriate if diagnostic uncertainty, progressive lesion growth, persistent sepsis, or concern for malignancy had remained.

## 13. Discussion

This review emphasizes hepatic infarction as a diagnostic-process problem rather than only as a rare pathologic diagnosis. For internists, the decisive issue is often not whether hepatic infarction exists in the abstract, but whether it is recognized early enough in the correct patient to prevent unnecessary drainage, biopsy, prolonged empirical antimicrobial therapy, delayed vascular imaging, or missed interventional complications. The revised pathway therefore starts from bedside probability and clinical trajectory, not from the radiologic label alone.

The literature also supports a broader vascular-inflammatory frame. Sepsis, shock, pylephlebitis, acute bland PVT, pancreatitis-associated thrombosis, and iatrogenic arterial injury can converge on the same final lesion morphology: a focal non-enhancing ischemic area. This convergence explains why the same hepatic lesion may sit at the boundary between acute medicine, hepatology, infectious diseases, surgery, interventional radiology, and radiology. It also explains why sterile infarction and secondarily infected infarction must be actively separated, because their management differs substantially.

Toll-like receptors (TLRs) are relevant to the broader biology of liver injury and repair, particularly after partial hepatectomy. Experimental and translational data indicate that innate immune signaling through TLR-related pathways can modulate priming of hepatocytes, cytokine signaling, Kupffer-cell activation, and the balance between regeneration and inflammatory injury. This does not imply that TLRs are a direct diagnostic or therapeutic target in routine hepatic infarction care. Rather, it reinforces a general principle: ischemic, infectious, inflammatory, and reparative pathways overlap in the liver, and the clinical phenotype of a focal lesion may reflect both vascular injury and the host response to tissue necrosis.

A final point concerns evidence quality and clinical authority. Most data on adult hepatic infarction come from case reports, small series, radiology reviews, and extrapolation from vascular liver disease guidance. The proposed pathway has not been prospectively validated and should not be interpreted as a guideline-level algorithm. It is a conceptual framework for organizing bedside reasoning, choosing appropriate imaging, avoiding premature invasive procedures, and prompting timely multidisciplinary discussion. Local resources, patient stability, renal function, contrast contraindications, procedural risk, and specialist availability must guide individual decisions.

## 14. Limitations

This review has important limitations. Hepatic infarction is rare, and the literature is dominated by case reports, small case series, radiology observations, transplant or interventional reports, and expert interpretation. Publication bias is likely, because unusual, severe, complicated, or visually striking cases are more likely to be reported than small or clinically silent infarcts.

No prospective diagnostic studies or validated clinical prediction models define the sensitivity or specificity of CT, MRI, CEUS, laboratory patterns, or combined pathways for adult hepatic infarction. The relative importance of etiologic settings cannot be estimated precisely from the available literature, and qualitative descriptors should be interpreted as clinical orientation rather than epidemiologic measurement.

The management suggestions in this article therefore cannot be considered evidence-based recommendations in a strict sense. They are intended to support cautious diagnostic reasoning, antimicrobial and procedural stewardship, and multidisciplinary communication until better prospective data become available.

## 15. Conclusions

Hepatic infarction is a rare but clinically significant form of focal ischemic liver injury. It is most often recognized when systemic hypoperfusion, splanchnic vasoconstriction, microvascular dysfunction, or local vascular compromise occur in combination. The condition is probably under-reported because many patients are acutely ill, lesions are nonspecific, and imaging may mimic abscess or malignancy.

A structured internist-led pathway is useful because no dedicated guideline exists. The proposed approach emphasizes bedside probability, contrast imaging, vascular assessment, key mimics, conservative management when appropriate, selective use of invasive procedures, and planned follow-up. Greater awareness among internists may improve diagnostic accuracy, trigger timely multidisciplinary care, and prevent unnecessary biopsy, drainage, surgery, or prolonged antimicrobial exposure.

### Key Practical Messages for Internists

- In hospitalized adults, hepatic infarction should be considered when a new focal hepatic lesion appears during sepsis, shock, vasopressor exposure, thrombosis, malignancy, vasculitis, transplantation, or after hepatobiliary/interventional procedures.
- The first bedside question is not “abscess or cancer?” but “does the clinical context and lesion geometry suggest focal ischemia?”
- A peripheral wedge-shaped or geographic non-enhancing lesion, especially with high lactate dehydrogenase (LDH) or abrupt aminotransferase rise, should prompt vascular-ischemic reasoning and review of arterial and portal venous flow.
- Drainage or biopsy should not be automatic. In typical infarction, conservative management and scheduled imaging follow-up may be safer than invasive procedures.
- Persistent fever, bacteremia, gas, enlarging fluid collection, rim enhancement, or clinical deterioration should shift the frame toward secondary infection of infarcted tissue and possible drainage.
- Acute pancreatitis, pylephlebitis, and acute bland portal vein thrombosis are uncommon but important contexts in which hepatic infarction or infarction-like lesions may occur.
- Early discussion with radiology, hepatology, infectious diseases, interventional radiology, surgery, or transplant teams is indicated when there is vascular occlusion, pseudoaneurysm, biliary ischemia, liver failure, or diagnostic uncertainty.

## Figures and Tables

**Table 1:** Diffuse hypoxic liver injury versus focal hepatic infarction: practical distinctions for internists. Supporting references for Table 1 include recent reviews and the imaging literature on hypoxic/ischemic liver injury and hepatic perfusion physiology [1,2,3,4,10,11,12,13].

Domain	Diffuse Hypoxic Liver Injury	Focal Hepatic Infarction
Dominant mechanism	Global reduction in oxygen delivery or utilization, usually during cardiac failure, respiratory failure, shock, or severe hypoxemia.	Regional parenchymal necrosis caused by focal arterial, portal venous, combined macrovascular, or microvascular perfusion failure.
Typical clinical context	Hemodynamic collapse, hypoxemia, low cardiac output, septic or cardiogenic shock.	Sepsis or shock plus local vascular compromise; hepatic artery thrombosis/pseudoaneurysm; portal vein thrombosis; transplantation; hepatobiliary or pancreatic procedures; hypercoagulability; vasculitis.
Laboratory pattern	Abrupt and often marked AST/ALT and LDH increase; bilirubin may be initially limited; improvement may follow restoration of perfusion.	Variable AST/ALT and LDH elevation; cholestasis may appear if biliary ischemia or arterial compromise is present; inflammatory markers may reflect the underlying illness rather than infection of the lesion.
Imaging pattern	Often no discrete focal lesion; imaging may be nonspecific unless another process coexists.	Peripheral wedge-shaped, geographic, segmental, or vascular-distribution non-enhancing lesion on multiphasic CT/MRI/CEUS.
Main diagnostic pitfall	Attributing marked aminotransferase elevation to primary hepatitis when the driver is circulatory or oxygen-delivery failure.	Mistaking a sterile infarct for abscess, metastasis, hematoma, or postoperative collection; assuming patent main vessels exclude microvascular ischemia.
Management implication	Restore perfusion and oxygenation and treat the underlying systemic cause.	Treat the underlying cause, assess arterial and portal venous patency, avoid automatic drainage/biopsy when imaging is typical, and monitor for secondary infection or actionable vascular lesions.

**Table 2:** Main etiologic settings of adult hepatic infarction and practical clues for internists. Supporting references for Table 2 include clinicopathologic studies, vascular liver disease guidance, the sepsis–immunothrombosis literature, the interventional/transplant literature, and reports on acute pancreatitis, pylephlebitis, and portal vein thrombosis [5,6,7,8,9,14,15,16,17,18,19,20,21,22,23,24,25,26,27]. Qualitative terms in this table are intended to orient bedside reasoning. They should not be interpreted as incidence estimates, pooled proportions, or mutually exclusive epidemiologic categories.

Etiologic Setting	Typical Mechanism	Clinical Clues	Immediate Practical Question
Systemic shock or sepsis	Hypoperfusion, splanchnic vasoconstriction, endothelial injury, immunothrombosis, DIC or sinusoidal stasis	Septic/cardiogenic shock, vasopressor exposure, lactate elevation, abrupt AST/ALT and LDH rise	Is the lesion a sterile ischemic infarct or secondarily infected necrosis?
Hepatic arterial compromise	Thrombosis, embolism, dissection, pseudoaneurysm, post-embolization ischemia	Recent procedure, transplant, arterial catheterization, bleeding, biliary ischemia, cholestasis	Is there an actionable arterial lesion requiring interventional radiology or surgery?
Portal venous compromise	Bland PVT, pylephlebitis, mesenteric thrombosis, local inflammatory thrombosis	Intra-abdominal infection, pancreatitis, malignancy, thrombophilia, bowel inflammation	Is anticoagulation, source control, antibiotics, or vascular consultation needed?
Iatrogenic or postoperative injury	Surgery, ablation, biopsy, biliary intervention, TACE/embolization, pancreatic surgery	Temporal relation with hepatobiliary/pancreatic procedure, new pain, fever, cholestasis, collection	Is the finding expected post-procedural ischemia or a complication needing treatment?
Prothrombotic or systemic disease	Cancer-associated thrombosis, antiphospholipid syndrome, vasculitis, DIC, trauma	Cancer, autoimmune disease, thrombophilia, vascular symptoms, thromboses elsewhere	Is the infarct the presenting clue of systemic vascular disease?
Toxic or vasospastic precipitants	Arterial vasospasm or thrombosis, often in combination with other risk factors	Cocaine, synthetic cannabinoids, severe vasoconstrictive states	Is there an exposure that changes recurrence prevention and counseling?

**Table 3:** Common diagnostic errors to avoid at the bedside.

Diagnostic Error	Why It Matters	Practical Safeguard
Mistaking every febrile non-enhancing lesion for abscess.	Sepsis may be the context in which sterile infarction occurs; unnecessary drainage can add bleeding or infectious risk.	Correlate lesion geometry, enhancement pattern, cultures, bacteremia persistence, gas, rim-enhancing collection, and clinical trajectory.
Mistaking a wedge-shaped infarct for metastasis or primary liver cancer.	Mass-like language can trigger unnecessary biopsy or oncologic work-up.	Review whether the lesion follows a vascular distribution and whether it remains persistently non-enhancing across phases.
Assuming patent main vessels exclude ischemia.	Sepsis-associated endothelial dysfunction, immunothrombosis, sinusoidal stasis, and vasopressor-treated shock may cause microvascular ischemia.	Interpret vascular patency together with hemodynamics, LDH/AST/ALT trajectory, coagulation, and lesion morphology.
Overlooking post-procedural arterial injury or pseudoaneurysm.	Actionable vascular complications can be missed if the lesion is treated only as infection or tumor.	Ask specifically about recent biopsy, biliary intervention, embolization, ablation, surgery, or transplantation and request vascular review when appropriate.
Proceeding directly to biopsy or drainage despite typical infarction.	Infarcted tissue can be nondiagnostic and fragile patients may be harmed.	Prefer conservative management and short-interval imaging when the clinical-imaging pattern is typical and there is no drainable infected collection or malignancy concern.

**Table 4:** Practical differential diagnosis of a new focal hepatic lesion in an acutely ill adult.

Diagnosis	Imaging Pattern	Clinical/Laboratory Clues	Pitfall to Avoid
Hepatic infarction	Peripheral wedge-shaped or geographic non-enhancing area; vascular distribution; possible capsular contact	Shock, sepsis, thrombosis, vasopressors, recent procedure, high LDH, abrupt AST/ALT rise	Do not assume all febrile non-enhancing lesions are abscesses.
Abscess	Rim enhancement, liquefaction, possible gas, diffusion restriction; may be round or multiloculated	Persistent fever, bacteremia, inflammatory markers, source of infection	Do not drain a sterile infarct only because fever is present in sepsis.
Malignancy/metastasis	Mass-like morphology; arterial enhancement or washout depending on tumor type; growth over time	Cancer history, tumor markers, systemic symptoms	Do not biopsy before adequate multiphasic imaging when geometry suggests ischemia.
Hematoma/postoperative collection	Variable density/signal according to blood products; relation to trauma/procedure	Recent surgery, anticoagulation, falling hemoglobin	Do not miss arterial pseudoaneurysm or active bleeding.
Ischemic cholangiopathy	Biliary strictures, cholestasis, biliary casts or ductal abnormalities	Transplant, arterial injury, shock, rising ALP/GGT	Do not dismiss cholestasis when aminotransferases are improving.

**Table 5:** Bedside checklist for internists when hepatic infarction is suspected.

Step	Question	Action
1. Recognize context	Is there shock, sepsis, thrombosis, cancer, transplant, vasopressors, or a recent procedure?	Frame the lesion as potentially vascular-ischemic before labeling it abscess or tumor.
2. Review labs	Is there abrupt AST/ALT or LDH rise, lactate elevation, cholestasis, coagulopathy?	Correlate timing of liver tests with hemodynamic and infectious events.
3. Characterize lesion	Is it wedge-shaped/geographic and non-enhancing?	Request or review multiphasic CT, MRI, CEUS, or Doppler/vascular imaging.
4. Search for vessels	Is there arterial thrombosis/pseudoaneurysm/dissection or PVT?	Discuss with radiology and interventional teams if actionable.
5. Separate sterile from infected	Is there gas, rim collection, persistent bacteremia, fever, or deterioration?	Escalate antibiotics, source control, and possible drainage only when infection is likely.
6. Avoid automatic biopsy	Is malignancy still plausible after adequate imaging?	Reserve biopsy for persistent diagnostic uncertainty or atypical evolution.
7. Plan follow-up	Is the patient stable and lesion typical?	Use CT, MRI, or CEUS to document expected evolution.

**Table 6:** Suggested multidisciplinary decision points.

Specialist/Team	When to Involve	Potential Contribution
Radiology	Any diagnostic uncertainty; need for multiphasic review or comparison	Confirm lesion geometry, enhancement behavior, vascular patency, and mimics.
Interventional radiology	Pseudoaneurysm, active bleeding, treatable arterial lesion, infected collection	Embolization, drainage when appropriate, vascular procedures.
Hepatology	PVT, ischemic cholangiopathy, liver failure, complex vascular liver disease	Anticoagulation strategy, cholangiopathy assessment, transplant-level referral when needed.
Infectious diseases/microbiology	Persistent bacteremia, pylephlebitis, secondary infected infarct	Antimicrobial selection and duration, source-control planning.
Surgery/transplant team	Postoperative or transplant vascular complication, biliary ischemia, liver failure	Assessment of surgical or transplant-specific interventions.
Hematology/rheumatology	Unexplained thrombosis, APS, vasculitis, cancer-associated thrombosis	Prothrombotic and inflammatory work-up and long-term prevention.

## Data Availability

No new data were created or analyzed in this study. Data sharing is not applicable to this article.
